# The clinical significance of pyogenic liver abscess after transarterial chemoembolization or microwave ablation on malignant liver tumors: A retrospective study

**DOI:** 10.1097/MD.0000000000039625

**Published:** 2024-09-13

**Authors:** Dong Yang, Dongyu Hu, Jing Hui, Zifeng Liu

**Affiliations:** a Oncology Department, Affiliated Hospital of Jining Medical University, Jining, Shandong, P. R. China; b Shandong University of Traditional Chinese Medicine, Jinan City, Shandong, P. R. China; c Oncology Department, Jining NO. 1 People’s Hospital, Jining, Shandong, P. R. China.

**Keywords:** microwave ablation, progression-free survival, pyogenic liver abscess, Sphincter of Oddi manipulation, transarterial chemoembolization

## Abstract

Pyogenic liver abscess (PLA) is a rare but severe complication of interventional therapy that has been little studied. We aimed to find the risk factors for PLA after transarterial chemoembolization (TACE) or microwave ablation (MWA), further explore its clinical significance and summarize our experience with its treatment. Twenty-two patients with PLA and 118 randomly selected patients without PLA after TACE/MWA were enrolled. Logistic regression was used to analyze risk factors, a nonparametric test was used to compare recovery duration, the log-rank test was used to compare progression-free survival, and Spearman correlation coefficient was calculated between the time from fever to drainage and the total duration of fever. The disease process and treatment were summarized. Sphincter of Oddi manipulation increased the risk of PLA by 70.781-fold. The PLA group took longer to recover (36.56 ± 16.42 days) than the control group (5.54 ± 4.33 days), and had a shorter progression-free survival. *Escherichia coli* was the major pathogenic bacterium, and multidrug resistance was found in 8 patients with *E coli* or *Enterococcus faecium*. The time from fever to drainage was 15.89 ± 13.78 days, which was positively correlated with the total duration of fever (24.29 ± 18.24 days). Overall, 18 patients recovered, and 4 patients died of PLA, for a mortality rate of 18.18%. The fever of 10 patients (45.45%) was controlled by cefoperazone sodium and sulbactam sodium or piperacillin sodium and tazobactam sodium; the fever of 7 patients (31.81%) was controlled by imipenem and cilastatin sodium; and the fever of 3 patients (13.63%) was controlled by tigecycline. Sphincter of Oddi manipulation is a high-risk factor for PLA after TACE or MWA. PLA can accelerate cancer progression and even lead to death. *E coli* was the major pathogenic bacterium, and multidrug resistance was most common in *E coli* and *E faecium*. Timely drainage and appropriate antibiotics are the key primary measures for treating PLA. Cefoperazone sodium and sulbactam sodium or piperacillin sodium and tazobactam sodium is a good choice for the first treatment of PLA, especially before pathogenic bacteria are identified. With the emergence of drug resistance, imipenem and cilastatin sodium, and tigecycline can be used for posterior treatment.

## 1. Introduction

Malignant liver tumors include primary and metastatic tumors, and surgical resection is the primary treatment.^[1,2]^ For some patients who are not suited for surgery because of poor physical condition, advanced clinical stage, special location of tumors, and so on, interventional therapies are usually adopted because of their advantages of minimal trauma and quick recovery.

Transarterial chemoembolization (TACE) and microwave ablation (MWA) are commonly used interventional therapies for malignant liver tumors. TACE can be performed using a mixture of chemotherapeutic agents and lipiodol in tumor-feeding arteries to control tumors.^[3]^ It is the first choice for large or multifocal hepatocellular carcinoma without macrovascular invasion or extrahepatic metastasis.^[4,5]^ MWA is a local treatment that can quickly raise the temperature of the tumor and generate a microwave field, resulting in coagulated necrosis.^[6,7]^ To ensure the safety of TACE and MWA, their complications have been widely studied.

Pyogenic liver abscess (PLA) is a rare complication of TACE or MWA, with an incidence of approximately 1%, but it is very troublesome and even life-threatening.^[8]^ Because of its low incidence, it is difficult to collect enough cases for research. Few studies have attempted to explore the possible risk factors for PLA, but without further research.^[9]^ Therefore, it is necessary to better understand the clinical significance of PLA in cancer patients undergoing TACE or MWA.

In this study, we further analyzed the risk factors for PLA after TACE or MWA. Importantly, we analyzed the effects of PLA on cancer progression for the first time to elucidate the potential negative effects of PLA on cancer patients. We also summarized and analyzed 22 patients’ pathogenic bacteria, multidrug resistance (MDR), disease processes, and treatment measures for PLA. We hope that this study will further our understanding of PLA in cancer patients after TACE or MWA, help clinicians realize the great harm PLA can cause to cancer patients, and provide a theoretical basis for making the correct choices to prevent and treat PLA.

## 2. Material and methods

### 2.1. Patient collection

This study followed the principles of the Declaration of Helsinki, and the study protocol was approved by the Ethics Committee of the Affiliated Hospital of Jining Medical University (reference number: 2021-10-C002). Due to the retrospective nature of the study, patient consent for inclusion was waived.

Patients with malignant liver tumors who underwent TACE or MWA in the hospital between May 2014 and August 2023 were reviewed. All patients were diagnosed by pathology or a typical enhancement pattern on computed tomography (CT) or magnetic resonance imaging according to the American Association for the Study of Liver Disease Guidelines. Twenty-two patients with PLA after TACE or MWA made up the PLA group. Among the many patients without PLA after TACE or MWA, we randomly selected 118 patients as the control group.

### 2.2. MWA procedure

The criteria to undergo MWA treatment were as follows: Eastern Cooperative Oncology Group performance status ≤ 2, international normalized ratio ≤ 1.5, platelet count ≥ 50,000/mL, and Child-Pugh class A or B. After agreement by the patients and their immediate family, who signed the informed consent, MWA operations were performed with CT guidance under general anesthesia using a saline-cooled antenna (211018, Vison-China Medical Devices R&D Center, Nanjing, China). All procedures were performed in sterile environments by experienced doctors.

### 2.3. TACE procedure

The criteria for receiving TACE treatment were as follows: Eastern Cooperative Oncology Group performance status ≤ 2, international normalized ratio ≤ 1.5, platelet count ≥ 50,000/mL, and Child-Pugh class A or B. Patients with a blocked main portal vein were excluded. After agreement by the patients and their immediate family, who signed the informed consent, TACE operations were performed with a digital angiography system (Philips, Amsterdam, Netherlands) under local anesthesia. The catheter was inserted into the hepatic artery through percutaneous femoral artery puncture, and after superselective cannulation was applied to the branch of the hepatic artery of the tumor, the patient was perfused with chemotherapy drugs and lipiodol. All procedures were performed in sterile environments by experienced doctors.

### 2.4. Diagnosis of PLA

The diagnostic criteria for PLA were as follows: enhanced CT image showing a peripheral enhancing rim around the ablation zone accompanied by any of 3 conditions: (1) fever higher than 38.5 °C for at least 3 days without other causes; (2) pathogenic bacteria in the aspirate or blood samples; and (3) repeated chills and fever.^[10]^

### 2.5. Puncture and drainage of the PLA

After agreement by the patients and their immediate family members, who signed the informed consent, puncture operations were performed with CT guidance under local anesthesia. After a puncture needle with a drainage tube was placed into the abscess cavity simultaneously, the puncture needle was withdrawn, and the drainage tube was left in the abscess cavity.

### 2.6. Follow-up

In the recovery period after PLA, an imaging examination was performed according to the state of illness. After the PLA was cured, follow-up imaging was performed every 3 to 6 months. The time to progression was calculated from TACE or MWA to cancer progression. The follow-up deadline for this study was April 2024. The recovery duration was timed from the MWA or TACE procedure to leaving the hospital or to receiving other antitumor therapies.

### 2.7. Statistical analysis

Continuous variables are expressed as mean ± standard deviation, and SPSS 27.0 (IBM Corp., Armonk, NY) was used for statistical analysis. Differences in continuous variables between the 2 groups were analyzed using a nonparametric test for nonnormally distributed variables. Logistic regression analysis was used to analyze risk factors for PLA. Spearman analysis was used for correlation analysis. The log-rank test was used to analyze differences in progression-free survival (PFS) between the 2 groups. Kaplan–Meier curves were plotted. Statistical significance was set at *P* < .05.

## 3. Results

### 3.1. Risk factors associated with PLA

**Figure 1.**
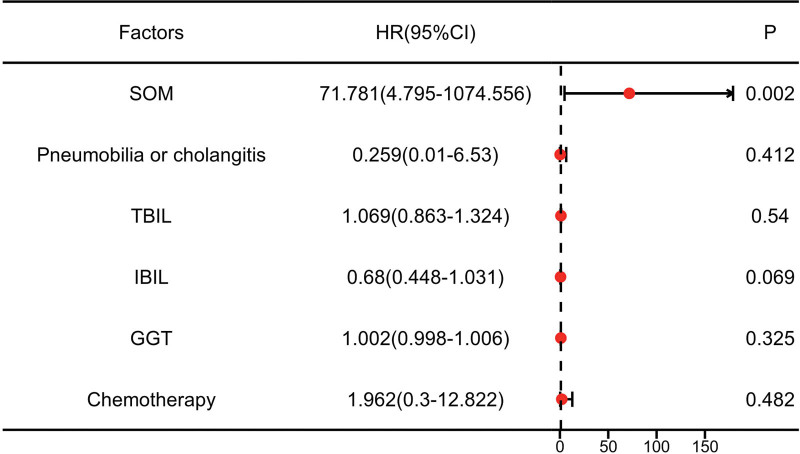
Analysis of risk factors for PLA. The forest plot of the multivariate logistic regression results showed correlations between sphincter of Oddi manipulation (SOM), pneumobilia or cholangitis, total bilirubin (TBIL), indirect bilirubin (IBIL), gamma-glutamyl transferase (GGT) and chemotherapy in patients with PLA. Statistical significance was set at *P* < .05.

The basic information of the 22 patients with PLA after TACE or MWA is shown in Table 1. Fifteen (68.18%) patients accepted Sphincter of Oddi manipulation (SOM), which can destroy the sphincter of Oddi; 9 (40.91%) patients had pneumobilia, and 1 (4.55%) had cholangitis. As shown in Table 2, univariate logistic regression analysis showed that SOM and pneumobilia or cholangitis were both positively correlated with a high risk of PLA (*P* < .001, *P* < .001); chemotherapy history was also positively correlated with a high risk of PLA (*P* = .04); gamma-glutamyl transferase (GGT) were correlated with a high risk of PLA(*P* = .021); high total bilirubin (TBIL) and indirect bilirubin (IBIL) were correlated with a low risk of PLA (*P* = .004, *P* < .001). Multivariate logistic regression analysis showed that only SOM was an independent risk factor for PLA, increasing the risk by 70.781-fold (Fig. 1, *P* = .002). Chemotherapy history, GGT, TBIL, and IBIL were not correlated with the risk of PLA.

**Table 1:** Basic information of 22 cases with PLA after TACE or MWA.

Case	Gender/age	Cancer	SOM	Pne or cho	TACE/	Bacteria	MDR	Dra	Out
					MWA				
1	Female/62	CHC	Yes	pne	TACE	*Enterobacter cloacae*	neg	Yes	rec
2	Male/66	PLC	No	No	TACE	*Escherichia coli*	neg	Yes	rec
3	Male/68	PC	Yes	No	MWA	*Klebsiella pneumoniae*	neg	Yes	rec
4	Female/47	LC	No	cho	MWA	*Escherichia coli* + *Enterococcus faecium*	pos	Yes	rec
5	Male/56	AC	Yes	pne	MWA	*Enterobacter cloacae* + *Enterococcus faecium*	pos	Yes	rec
6	Male/62	DC	Yes	pne	MWA	*Escherichia coli*	pos	Yes	rec
7	Male/58	CHC	Yes	pne	MWA	*Escherichia coli*	neg	Yes	rec
8	Female/51	EGJC	Yes	No	MWA	*Escherichia coli*	neg	Yes	rec
9	Female/47	PLC	Yes	No	TACE	*Escherichia coli* + *Enterococcus faecium*	pos	Yes	dead
10	Male/39	AC	Yes	pne	MWA	*Unknown*	neg	Yes	rec
11	female/52	CHC	Yes	pne	TACE	*Escherichia coli*	pos	Yes	rec
12	Male/67	CHC	Yes	pne	MWA	*Escherichia coli*	neg	No	rec
13	Male/47	CC	No	No	TACE	*Unknown*	neg	Yes	rec
14	Female/46	AC	Yes	pne	MWA	*Escherichia coli*	pos	Yes	rec
15	Male/59	CHC	Yes	No	MWA	*Escherichia coli* + *Enterococcus faecium*	pos	No	dead
16	Male/51	PLC	No	No	TACE	Unknown	neg	No	rec
17	Male/70	CHC	Yes	No	TACE	*Escherichia coli* + *Enterococcus faecium*	pos	Yes	dead
18	Female/56	PLC	No	No	TACE	Unknown	neg	No	rec
19	Male/52	PLC	No	No	TACE	Unknown	neg	No	rec
20	Female/53	DC	Yes	No	TACE	*Enterococcus faecium*	neg	Yes	rec
21	Male/61	PLC	No	No	TACE	*Escherichia coli*	neg	Yes	rec
22	Male/59	CHC	Yes	pne	TACE	*Escherichia coli*	neg	Yes	dead

AC = ampullary carcinoma, ant = anti-inflammation, CC = colon cancer, CHC = cholangiocarcinoma, cho = cholangitis, DC = duodenal carcinoma, dra = drainage, EGJC = esophagogastric junction carcinoma, LC = lung cancer, MDR = multidrug resistance, neg = negative, out = outcome, PC = pancreatic cancer, PLC = primary liver cancer, pne = pneumobilia, pos = positive, rec = recovery, SOM = Sphincter of Oddi manipulation.

**Table 2:** Univariate logistic regression of risk factors of PLA.

Clinical parameters	HR	95% CI	*P*
Age	0.986	0.944–1.03	.534
Gender	0.53	0.202–1.387	.196
Diabetes	1.65	0.541–5.038	.379
hypertension	1.283	0.482–3.42	.618
SOM	267.429	30.859–2317.602	<.001***
Chemotherapy history	2.616	1.046–6.543	.04*
Pneumobilia/cholangitis	97.5	11.474–828.494	<.001***
Globulin	0.985	0.903–1.075	.74
GGT	1.002	1.00–1.004	.021*
TBIL	0.87	0.791–0.958	.004**
DBIL	0.987	0.893–1.092	.804
IBIL	0.696	0.586–0.828	<.001***

DBIL = direct bilirubin, GGT = gamma-glutamyl transferase, IBIL = indirect bilirubin, SOM = sphincter of Oddi manipulation, TBIL = total bilirubin. *P* < .05 was statistically significant.

* *P* < 0.05, **P < 0.01, ***P < 0.001.

### 3.2. Effects of PLA on the recovery and prognosis of patients after TACE or MWA

**Figure 2.**
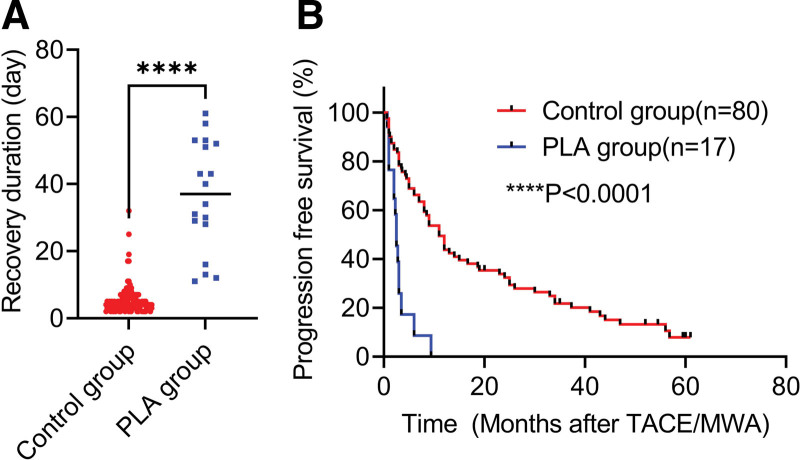
Analysis of the recovery and prognosis of patients with PLA. (a) Difference in recovery duration between the control and PLA groups. (b) Differences in progression-free survival (PFS) between the control and PLA groups. Statistical significance was set at *P* < .05.

In the control group, the recovery duration after TACE or MWA was 5.54 ± 4.33 days, ranging from 2 to 32 days. In the PLA group, 4 patients (18.18%) died of PLA, and the recovery duration of the other 18 patients was 36.56 ± 16.42 days, ranging from 11 to 61 days, about 7 times that of the control group with significant difference (Fig. 2a, *P* < .001). In the control group, the 1-, 2-, and 3-year PFS rates of the 80 patients were 47.50%, 37.50%, and 28.75%, respectively, with a median PFS (mPFS) of 11 months. In the PLA group, except the 4 patients who died of PLA, 11 patients with successful follow-up experienced cancer progression within 10 months, and the 1-year PFS rate was 0, with a mPFS of 2.5 months. The PFS of the PLA group was significantly worse than that of the control group (Fig. 2b; *P* < .0001).

### 3.3. Treatment measures for PLA

**Figure 3.**
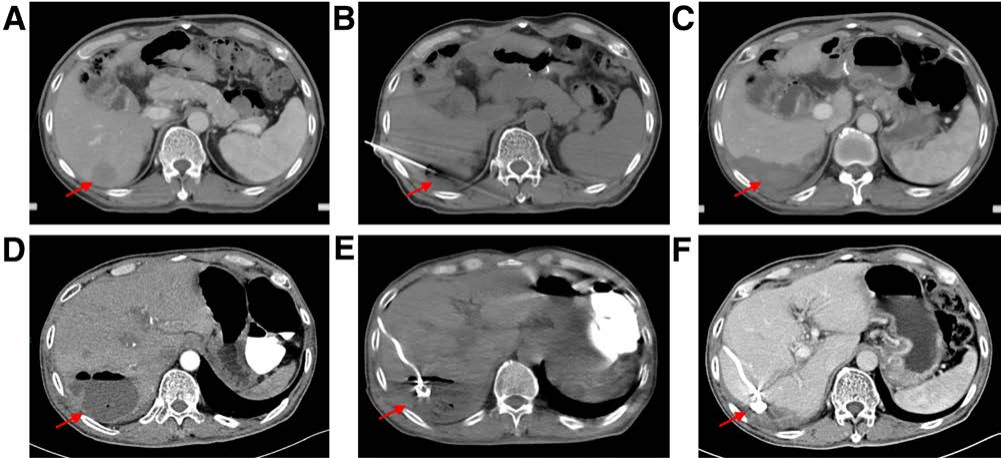
CT images of PLA. The tumor lesion before (a), during (b), and after (c) MWA. PLA cavity before (d), during (e), and after (f) treatment. The arrow indicates the lesion.

**Figure 4.**
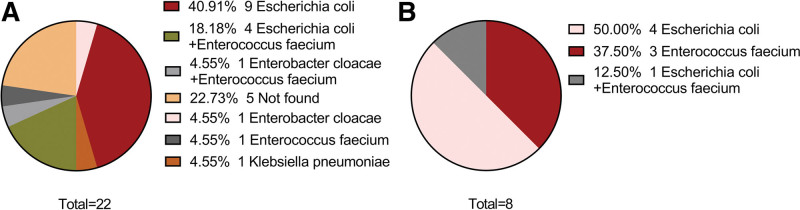
Distribution of pathogenic bacteria. (a) Distribution of pathogenic bacteria in 22 patients with pyogenic liver abscess (PLA). (b) Distribution of pathogenic bacteria in 8 patients with multidrug resistance (MDR).

**Figure 5.**
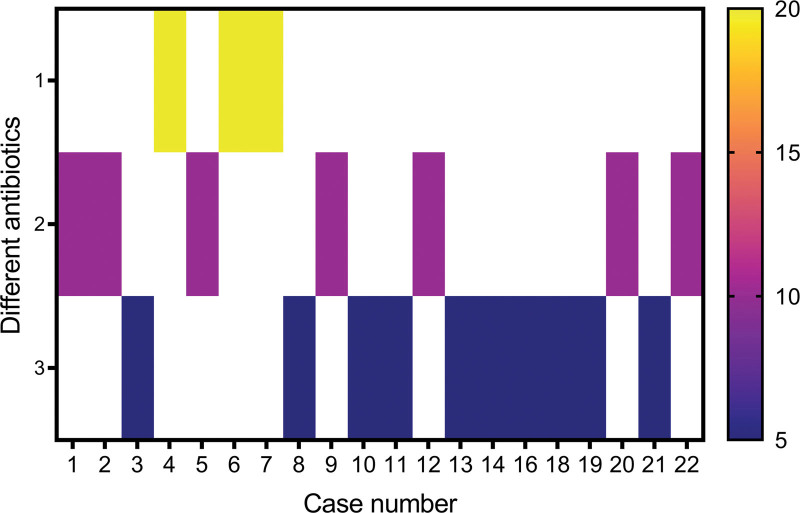
Antibiotics used in 20 patients with controlled fever. On the Y-axis, 1, tigecycline; 2 imipenem and cilastatin sodium (ICS); 3 cefoperazone sodium and sulbactam sodium (CSSS) or piperacillin sodium and tazobactam sodium (PSTS). The numbers on the X-axis represent 20 patients.

**Figure 6.**
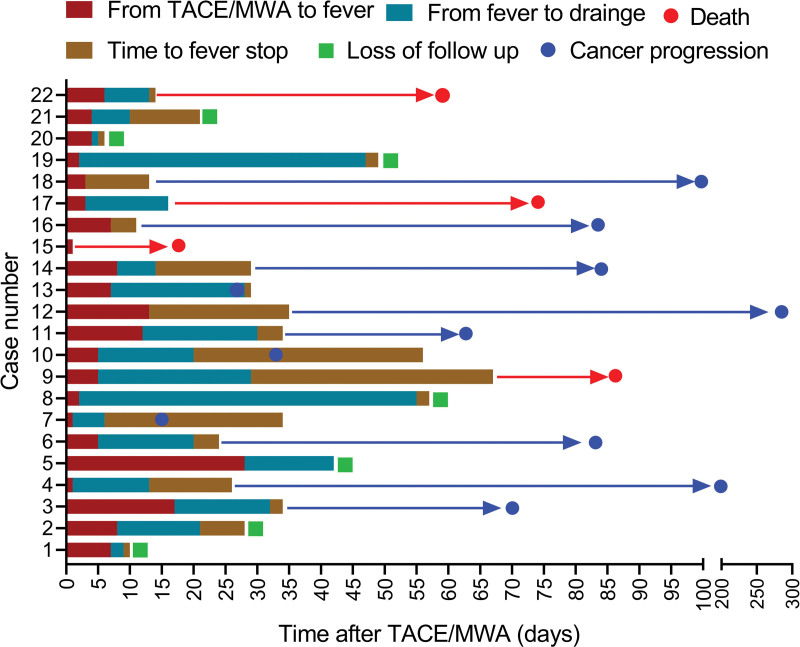
Histogram of disease process. The histogram shows different processes for the 22 PLA patients, including 18 patients who recovered from PLA, 4 patients who died of PLA, and 7 patients who were lost to follow-up.

**Figure 7.**
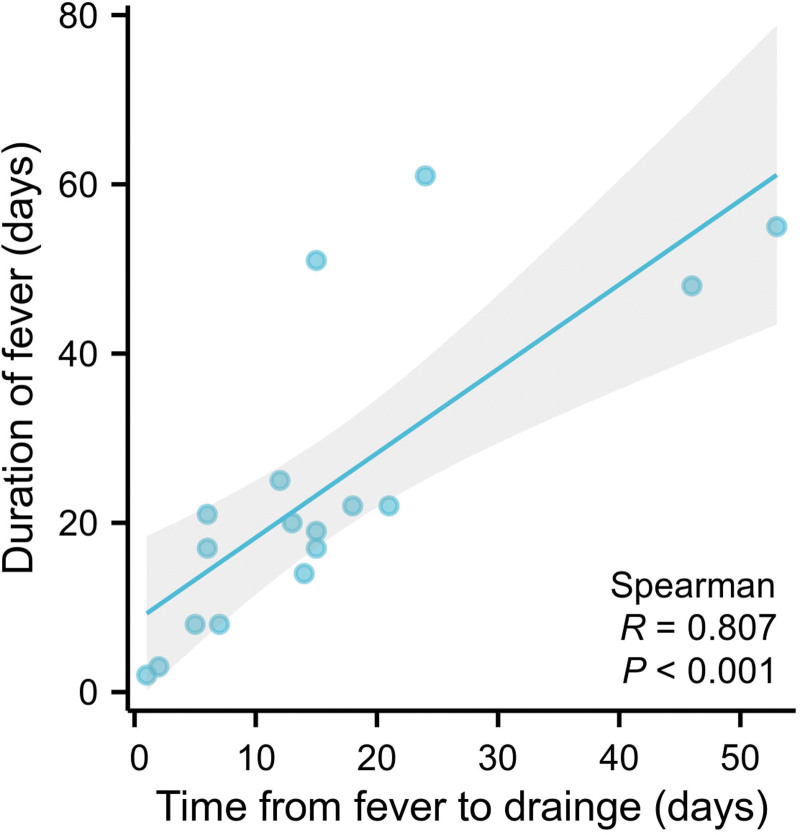
Correlation between the time from fever to drainage and fever duration. Spearman analysis revealed a correlation between the time from fever to drainage and fever duration. Statistical significance was set at *P* < .05.

Drainage is an important therapeutic measure for treating PLA. After the drainage tube was placed into the PLA cavity with a liquid plane, pus was emptied, and the cavity gradually shrunk or even disappeared (Fig. 3). Effective anti-inflammatory treatment is also a key factor in controlling PLA. The number of patients with *Escherichia coli*, *E coli* + *Enterococcus faecium*, *E faecium*, *Enterobacter cloacae*, *Klebsiella pneumobiliae*, and *E cloacae* + *E faecium* were 9 (40.91%), 4 (18.18%), 1 (4.55%), 1 (4.55%), 1 (4.55%), and 1 (4.55%), respectively. No pathogenic bacteria were found in 5 (22.73%) cases (Fig. 4a). Bacteria with MDR were found in 8 patients, including 4 patients with *E coli* (50.00%), 3 patients with *E faecium* (37.50%) and 1 patient with *E coli* + *E faecium* (12.50%) (Fig. 4b). As shown in Figure 5, the fever of 10 patients (45.45%) was controlled by cefoperazone sodium and sulbactam sodium (CSSS) or piperacillin sodium and tazobactam sodium (PSTS); 7 patients (31.81%) developed resistance to CSSS or PSTS, and their fever was stopped after treatment of imipenem and cilastatin sodium (ICS); 3 patients (13.63%) developed resistance to both CSSS/PSTS and ICS, and their fever was stopped after treatment with tigecycline. Figure 6 shows that the time from TACE/MWA to the onset of fever was 6.78 ± 6.26 days, ranging from 1 to 28 days; the time from onset of fever to drainage was 15.89 ± 13.78 days, ranging from 1 to 53 days; the time from drainage to fever stop was 10.28 ± 11.93 days, ranging from 1 to 38 days; and the total duration of fever was 24.29 ± 18.24 days, ranging from 2 to 61 days. Four patients died of PLA, including 2 patients with uncontrolled fever and 2 patients with controlled fever (Table 1). Eleven patients experienced cancer progression <10 months after TACE or MWA, ranging from 21 to 287 days; 3 patients experienced cancer progression even during fever. Spearman analysis revealed that the time from the onset of fever to drainage and the total duration of fever tended to be positively correlated (*R* = 0.807) (Fig. 7, *P* < .001).

## 4. Discussion

PLA is rare, and it mainly occurs in elderly people, people with diabetes, and cancer patients. Pathogenic bacteria may arise from the biliary tract, intestinal tract, blood circulation, or penetrating trauma; *K pneumobiliae* is considered the main pathogenic bacteria.^[11–13]^ PLA induced by TACE or MWA in cancer patients is even more rare, and it has been little studied. In 2005, Kim et al reported that 13 out of 751 patients (1.7%) developed PLA after radiofrequency ablation for hepatocellular carcinoma, and biliary abnormalities prone to ascending biliary infection were positively associated with PLA.^[14]^ The clinical significance of PLA induced by TACE or MWA in cancer patients and a summary of their treatment experience have not yet been reported.

In this study, 15 out of 22 patients with PLA had undergone SOM, including biliary surgery, pancreaticoduodenectomy, and biliary stent implantation surgery, which can destroy the sphincter of Oddi; 10 patients had pneumobilia or cholangitis. *K pneumobiliae* has been the main pathogenic bacteria of conventional PLA reported previously,^[15]^ but here *E coli* was the main pathogenic bacteria of PLA induced by TACE or MWA, implying a unique mechanism. As all 4 types of bacteria found in 22 patients, namely, *E coli*, *E faecium*, *E cloacae*, and *K pneumobiliae*, were associated with the intestinal microbiome,^[16–19]^ PLA infection after TACE or MWA was mainly caused by ascending intestinal bacteria. Moreover, SOM, which can destroy the sphincter of Oddi, is considered an important reason for the upward movement of intestinal bacteria.^[20]^ Pneumobilia refers to the presence of air within the biliary system, often caused by an abnormal connection between the biliary and gastrointestinal tracts, leading to the upward movement of intestinal bacteria.^[21]^ It is noteworthy that all 9 cases in this study demonstrating pneumobilia had previously undergone SOM, further suggesting a possible link among SOM, pneumobilia and the upward movement of intestinal bacteria.

Bilirubin is the catabolic product of heme, which mainly results from hemoglobin degradation in red blood cells, including indirect bilirubin (IBIL) and direct bilirubin (DBIL).^[22]^ IBIL is usually decreased when hemoglobin and red blood cells are defective in patients with dystrophic anemia, especially in patients with malignant tumors.^[22–24]^ Serum IBIL has been negatively correlated with the inflammatory marker C-reactive protein and can play an anti-inflammatory role by inhibiting the infiltration of inflammatory cells and reducing the levels of proinflammatory cytokines.^[25,26]^ The above data show that low levels of TBIL and IBIL may be associated with a poor physical condition and susceptibility to infection. In this study, for the first time, univariate logistic regression analysis found that both TBIL and IBIL were negatively correlated with the risk of PLA, which is in line with the above roles of IBIL. GGT can be secreted by bile duct endothelial cells, and serum GGT can be increased in several conditions, including liver injury and cholangitis.^[27]^ Xu Wang reported that the serum GGT concentration is positively correlated with the inflammatory marker C-reactive protein.^[25]^ In this study, univariate logistic regression analysis also found that serum GGT was positively correlated with a greater risk of PLA, which is in line with previous reports on GGT. Chemotherapy can not only kill tumors but also worsen the physical condition of cancer patients, making the patient less able to resist infection. In this study, univariate logistic regression analysis found that chemotherapy significantly increased the risk of PLA. Diabetes is a disease characterized by high blood glucose that occurs when the pancreas is unable to produce enough insulin or when the body is unable to use insulin effectively. Because a hyperglycemic environment is conducive to bacterial growth and long-term hyperglycemia leads to decreased resistance, diabetes patients are prone to various infections.^[28]^ In this study, univariate logistic regression analysis found that a history of diabetes tended to come with a greater risk of PLA, but the correlation was not statistically significant, contradicting the report of Ming Yan.^[10]^ On the one hand, this discrepancy might be attributed to the limited number of patients in the 2 studies. On the other hand, this might be associated with the different levels of blood glucose in patients after different treatments. Finally, multivariate logistic regression analysis confirmed that only SOM significantly increased the risk of PLA. We think that although pneumobilia or cholangitis, chemotherapy history, GGT, TBIL, and IBIL did not show significant correlations with PLA in our multivariate logistic regression analysis, we should pay attention to them along with SOM before TACE or MWA in ordinary clinical work to make sure we are vigilant about PLA. This study showed that the time from TACE/MWA to the onset of fever was 6.78 ± 6.26 days, ranging from 1 to 28 days. These results imply that for patients at risk of PLA, the occurrence of PLA should always be considered within at least 28 days after TACE or MWA.

Although the mortality of PLA is estimated to be between 5.6% and 22%,^[29,30]^ the harm of PLA induced by TACE or MWA in cancer patients has not yet been specifically reported. To fully understand the harmful effects of PLA induced by TACE or MWA on cancer patients, we analyzed recovery and prognosis. We found that the recovery duration of the PLA group was 36.56 ± 16.42 days, ranging from 11 to 61 days, approximately 7 times that of the control group. With such a long recovery time, antitumor therapy was forbidden because of the poor physical condition of patients, which seriously delayed antitumor treatment and provided the chance for cancer progression. In this study, 4 patients died of PLA, for a mortality rate of 18.18%, which is in line with previous reports. Among them, 2 patients had uncontrollable fever, and although the fever stopped in the other 2 patients, due to the heavy burden PLA put on the patients’ bodies and the poor physical condition of the cancer patients, they died of organ failure. We lost 7 PLA patients to follow-up, and the other 11 PLA patients experienced cancer progression <10 months after TACE or MWA, with an mPFS of 2.5 months. These data specifically demonstrate the great harm PLA does to cancer patients, suggesting that for cancer patients with a high risk of PLA, it is necessary to fully weigh the benefits and risks before deciding whether to do TACE or MWA.

Drainage and appropriate antimicrobial treatment are the primary treatment strategies for PLA.^[8,31]^ Puncture at the right time is the key to successful drainage. Premature puncture of a completely liquefied abscess cannot successfully drain pus, and a late puncture delays treatment. Hence, a timely CT scan is necessary to evaluate liquefaction status to determine the right puncture time for PLA patients. In this study, the duration from the onset of fever to drainage was 15.89 ± 13.78 days, ranging from 1 to 53 days, suggesting that abscess drainage can be successfully completed as early as 1 day after fever, but the longest interval from fever to successful drainage is uncertain because some patients have delayed puncture and drainage for various reasons. Moreover, the duration from the onset of fever to drainage is positively correlated with the total fever duration, which suggests the key role of drainage in controlling PLA. During the treatment process, *E coli* and *E faecium* were pathogenic bacteria that cause MDR, in line with their strong production of virulence factors and susceptibility to drug resistance reported previously.^[32,33]^ This reminds us that special attention should be paid to the occurrence of MDR in PLA patients with *E coli* or *E faecium* and frequent blood cultures are needed. Hydrolyzing antibiotics by producing β-lactamases is an important drug resistance mechanism in bacteria. CSSS or PSTS contain β-lactamase inhibitors that can irreversibly inhibit β-lactamase and prevent the occurrence of drug resistance.^[34]^ ICS is a new type of β-lactam antibiotic that is stable against β-lactamase and thus has a stronger antibacterial effect than CSSS or PSTS.^[35]^ Tigecycline is a glycylcycline antibiotic with strong antibacterial effects that cannot be affected by multiple drug resistance mechanisms, including β-lactamase and target modification.^[36]^ For all 22 patients in this study, cephalosporins did not have a controlling effect on PLA, the fever of 10 patients was controlled after treatment with CSSS or PSTS, the fever of 7 patients was controlled by ICS after drug resistance of CSSS or PSTS, and the fever of 3 patients was controlled by tigecycline after drug resistance of CSSS or PSTS and ICS. This suggests that, before pathogenic bacteria are identified, CSSS or PSTS is a good choice for the first treatment of PLA, especially before pathogenic bacteria are identified. With the emergence of drug resistance, ICS and tigecycline can be used for posterior treatment. In addition, given that the pathogenic bacteria were mainly ascending intestinal bacteria, we hypothesize that emptying the intestines with laxatives and CSSS or PSTS treatment before TACE or MWA might prevent the occurrence of PLA, but this hypothesis still needs to be tested.

The limited number of cases is the main drawback of this study. Because of the very low incidence of PLA after interventional therapy, only 22 patients with PLA were enrolled in this study. This makes the results susceptible to the influence of accidental factors including economic situation, treatment compliance and so on, limiting the accuracy and reliability of our results. In addition, this was a single-center study, and the patients were all from surrounding areas, so the results have certain geographical limitations. A multicenter study with more patients will provide more reliable and universal results. In conclusion, a history of SOM is an independent risk factor and increased the risk of PLA by 70.781-fold. We should be vigilant about the occurrence of PLA within at least 28 days after TACE or MWA. PLA can cause great harm to cancer patients, including cancer progression and death. Therefore, for patients with a history of SOM, the pros and cons should be fully weighed before TACE or MWA. The pathogenic bacteria of PLA are mainly ascending intestinal bacteria, and patients with MDR strains, especially *E coli* and *E faecium*, should be vigilantly monitored. Timely drainage combined with proper antibiotics is key to good outcomes, and CSSS or PSTS is a good choice for controlling PLA, especially before pathogenic bacteria are identified. With the emergence of drug resistance, ICS and tigecycline can be used for posterior treatment according to drug sensitivity test.

## Author contributions

**Conceptualization:** Zifeng Liu.

**Data curation:** Dongyu Hu.

**Formal analysis:** Dongyu Hu.

**Writing – original draft:** Dong Yang.

**Writing – review & editing:** Jing Hui, Zifeng Liu.
